# Effects of Astragaloside IV on heart failure in rats

**DOI:** 10.1186/1749-8546-4-6

**Published:** 2009-04-02

**Authors:** Zhuanyou Zhao, Weiting Wang, Fang Wang, Kerui Zhao, Yingmei Han, Weiren Xu, Lida Tang

**Affiliations:** 1 State Key Laboratory of Pharmacokinetics and Pharmacodynamics, Tianjin Institute of Pharmaceutical Research, Tianjin 300193, PR China; 2 Department of Pharmacology, Tongji Medical College, Huazhong University of Science and Technology, Wuhan 430030, PR China; 3 Department of Pharmacy, Tianjin Medical College, Tianjin 300222, PR China

## Abstract

**Background:**

Astragaloside IV (ASI) in *Radix Astragali *is believed to be the active component in treating heart failure. The present study aims to examine the effects of ASI on cardiovascular parameters in long-term heart failure in rats.

**Methods:**

Using echocardiographic and haemodynamic measurements, we studied the effects of ASI on congestive heart failure (CHF) induced by ligation of the left coronary artery in rats.

**Results:**

ASI (0.1, 0.3 and 1.0 mg/kg/day) attenuated the decline of fractional shortening (FS). The peak derivatives of the left ventricle (LV) pressure (dp/dt) in ASI-treated groups significantly increased. Both LV internal diameters in diastole (LVIDd) and in systole (LVIDs) decreased significantly after ASI treatment (0.3 and 1.0 mg/kg/day). ASI (1.0 mg/kg/day) attenuated the decrease of LV systolic pressure (LVSP). ASI treatment inhibited compensatory hypertrophy of myocardial cells and lowered the number of apoptotic myocytes.

**Conclusion:**

ASI improved cardiac functions as measured by cardiovascular parameters.

## Background

Heart failure is a major cause of morbidity and mortality around the world and remains a serious outcome of myocardial infarction (MI). *Radix Astragali *(*Huangqi*) is a Chinese herbal medicine reported to improve the left ventricular function and mitigating the cardiac hypertrophy in heart failure patients [1-5]. Recently, increasing evidence shows that Astragaloside IV (ASI), which is a saponin, is the active ingredient in the treatment of heart failure [6-10]. ASI increased systolic and diastolic functions in acute heart failure of canine induced by pentobarbital sodium and improved the cardiac depressant effect induced by propanolol. Another study [11] further demonstrated a protective effect on the left ventricular modelling and ejection function in patients with congestive heart failure (CHF) after a two-week continuous administration of ASI. Furthermore, the antiapoptotic activities of ASI were discovered in some diseases such as myocarditis [12].

Recent studies showed that cardiac hypertrophy and apoptosis were correlated with the severity of CHF [13,14]. Cardiocyte apoptosis plays an important role in the remodelling process and in the transition from adaptive myocardial condition to end-stage cardiac failure. Cardiac apoptosis is a genetically programmed process executed by caspases, a family of ubiquitous cysteine proteases. Caspases are present in the cell as inactive pro-caspases, which are activated in response to apoptotic stimuli including myocardial pathological conditions (e.g. hypoxia, oxidation, and mechanical load), cytokines (e.g. TNF-α, IL-6 and ITF) and neuroendocrine factors (e.g. angiotensin II and NO) [15]. Gene families Bcl-2 and P53 also take part in the regulation of apoptosis [16]. However, the effects of ASI on heart failure have not been extensively elucidated.

The present study aims to examine the effects of ASI on cardiovascular parameters in long-term heart failure as an outcome of MI in rats.

## Methods

### Animals and reagents

Wistar rats weighting 228.3 ± 13.0 g (203–272 g) were housed at 22 ± 2°C in a temperature-regulated room equipped with an automatic 12:12 hour photoperiod and were provided with food and water. ASI is colourless needles crystallized from Astragaloside by ethyl acetate-ethanol, melting point 295–300°C, purity>95%. ASI was dissolved in 20% dimethylsulfoxide (<0.05%) and diluted with normal saline (NS) when studied *in vivo*. Quinapril was supplied by the Shanghai Institute of Pharmaceutical Industry (certificate no.051).

### Induction of MI and medications

MI in Wistar rats was induced by coronary ligation. Through a thoracotomy on the left side, the heart was exteriorized and extensive MI was induced by ligation of the left coronary artery according to Tonnessen *et al*. [17]. Animals in the sham group were subjected to the same surgical procedure, except that the coronary artery was not ligated. Echocardiography was performed three weeks later in the surviving rats anaesthetized by ether; those with heart failure according to echocardiographic criteria were randomly divided into five groups [18,19] where each group had ten animals. Group one (model) received NS; group two to four received ASI at 0.1, 0.5, 1.0 mg/kg respectively; group five received quinapril at 1.0 mg/kg. The animals in the sham group received NS. The treatment, which started three weeks after coronary ligation, was carried out via intravenous injection once a day for 14 days. The experiment lasted for five weeks.

### Echocardiography

We used a commercially available echocardiography (VIVID 3 Pro, GE, USA) equipped with a 10 MHz linear transducer. The animals, with shaved chests, were placed in supine position. The transducer was placed gently on the left. Two-dimensional (2D) images of the left ventricle (LV) were obtained in both parasternal long-axis and short-axis views at a frame rate of 130 Hz. M-mode tracings were recorded at the level of the papillary muscles with 2D guidance. LV wall thickness and cavity diameters were measured outside the infarcted area by M-mode, through the largest diameters of the ventricle. Each image consisted of 24 consecutive heart cycles and the focus area was 0–40 mm from the transducer. Fractional shortening (FS), in percentage, was calculated according to the following formula:



where LVIDd is the LV internal diameter in diastole, and LVIDs is the LV internal diameter in systole [20]. LV internal diameters were measured according to the guidelines of the American Society of Echocardiography [21]. All measurements were obtained on at least three beats. LV systolic wall stress = 1.36 × (aortic systolic pressure × LVIDs)/(IVSs + LVPWs), where IVSs is the interventricular septum thickness at end-systole and LVPWs is the posterior wall thickness at end-systole. The tracings were analyzed by one author (WTW) who had no prior knowledge of the group assignment.

### Haemodynamic measurements

An F-2 (0.7 mm in internal diameter) catheter with pressure-transducer was inserted through the right carotid artery into the aorta to determine the mean arterial blood pressure. The catheter was subsequently advanced further into the LV, and end-diastolic pressure (LVEDP) and systolic pressure (LVSP) were recorded. The transducer was connected to a polygraph (model 6300, Nihon Kohden, Japan) and a data acquisition workstation (model MP150, BIOPAC Systems, USA) to a computer running Acknowledge (version 3.7.1). LVEDP was read directly from the pressure curve as being the diastolic pressure immediately preceding the pressure rise associated with LV contraction. Peak positive and peak negative first derivatives of the LV pressure (+LVdp/dt and -LVdp/dt) were calculated with the Acknowledge software.

### Tissue sampling

After haemodynamic measurements, the rats were subsequently euthanized by excision of the heart. The atria and ventricles were sectioned, separated and weighted. The infarcted area (scar tissue) was excised with care from the non-infarcted LV to avoid contamination of viable myocardial tissue by necrotic tissue. The infarction size was estimated by weighing the excised scar tissue. The non-infarcted LV from the CHF rats was further sectioned by separating the tissue contiguous to the infarcted area (viable LV free wall) and the tissue distal to the infarcted area (interventricular septum). A piece of the apical part of the nonischemic interventricular septum was processed separately for histochemical analysis of apoptosis. The right tibia of each rat was isolated and its length measured [22]. Myocardial tissue from interventricular septum was microtomed into 5 μm sections. Apoptotic cell death was analyzed by terminal-deoxynucleotidyl transferase (TdT)-mediated dUTP-digoxigenin nick end labelling (TUNEL) method. The Bcl-2 associated X protein (Bax) related to apoptosis was determined with biotin SP-HRP.

### Statistical analysis

Data are expressed as mean ± standard deviation (SD). The statistical significance between groups was evaluated by a one-way analysis of variance (ANOVA) with SAS software (version 10.0, SAS Institute, USA). *P *values < 0.05 are considered to be statistically significant.

## Results

### Effects of ASI on the LV functions

Three weeks after coronary artery ligation, the FS of the animals in the model group markedly decreased and further decreased after the animals were given NS for two more weeks. The values of +dp/dt and -dp/dt decreased 36% and 48% respectively two weeks after NS administration. One week after the administration of ASI (0.1, 0.3 and 1.0 mg/kg/day), the decline of the FS was attenuated (*P *< 0.05, *P *< 0.001 and *P *< -0.001). Two weeks after the administration of ASI (at a dose of 1.0 mg/kg/day), the decline of the FS was attenuated (*P *< 0.05). The dp/dt in the ASI-treated groups (0.3 and 1.0 mg/kg/day) significantly increased compared with the model group (*P *< 0.05 and *P *< 0.01) (Tables 1 and 2).

**Table 1:** Effects of ASI on FS (%)

				After treatment	
Group	Dose (mg/kg/day)	Normal	Before treatment	1 week	2 weeks
Sham	-	73.0 ± 5.7	68.8 ± 6.5	70.9 ± 3.4	66.1 ± 8.0
Model	-	68.8 ± 10.1 (0.2193)	30.8 ± 5.0^+++^	26.1 ± 6.6^+++^	22.7 ± 7.6^+++^
ASI	0.1	72.8 ± 3.6 (0.2417)	28.9 ± 4.6 (0.4213)	34.2 ± 7.5* (0.0163)	27.4 ± 7.8 (0.2620)
ASI	0.3	72.1 ± 9.6 (0.3329)	28.8 ± 6.9 (0.3973)	41.5 ± 11.3***	28.8 ± 8.9 (0.1474)
ASI	1.0	68.4 ± 6.7 (0.9061)	31.7 ± 2.5 (0.7024)	42.9 ± 7.7***	35.8 ± 13.2** (0.0028)
Quinapril	1.0	70.1 ± 7.5 (0.7016)	30.2 ± 4.7 (0.7989)	42.4 ± 4.7***	31.4 ± 8.8* (0.0411)

Note: Values are mean ± SD in each group (*n *= 10). ^+++^*P *< 0.001: statistical significance over the sham group (comparison of group means after ANOVA). *P < 0.05, **P < 0.01, ***P < 0.001: statistical significances over the model group (comparison of group means after ANOVA). No statistical significance between ASI (1.0 mg/kg/day) and Quinapril treatments. Exact *P *values are available in brackets.

**Table 2:** Effects of ASI on +dp/dt and -dp/dt

Group	Dose (mg/kg/day)	+dp/dt (mmHg/s)	-dp/dt (mmHg/s)
Sham	-	3454 ± 774	3706 ± 517
Model	-	2216 ± 800^+++^	1922 ± 672^+++^
ASI	0.1	2459 ± 524 (0.3679)	2362 ± 610 (0.0710)
ASI	0.3	2838 ± 420* (0.0246)	2699 ± 425** (0.0021)
ASI	1.0	3066 ± 630** (0.0027)	2776 ± 578***
Quinapril	1.0	2847 ± 251* (0.0228)	2601 ± 310** (0.0065)

Note: Values are mean ± SD in each group (*n *= 10). ^+++^*P *< 0.001: statistical significance over the sham group (comparison of group means after ANOVA). *P < 0.05, **P < 0.01, ***P < 0.001: statistical significance over the model group (comparison of group means after ANOVA). No significance between ASI (1.0 mg/kg/day) and Quinapril treatments. Exact *P *values are available in brackets.

### Effects of ASI on LV dimensions

LV geometry in various groups was analyzed with echocardiography (Table 3). The findings demonstrated the progression of LV remodelling during a 5-week period after coronary artery ligation in the absence of treatment. Both LVIDd and LVIDs increased significantly and progressively three weeks after surgery. Both LVIDd and LVIDs decreased significantly two weeks after ASI treatment (0.3 and 1.0 mg/kg/day).

**Table 3:** Effects of ASI on LV dimensions

					After treatment	
	Group	Dose (mg/kg/day)	Normal	Before treatment	1 week	2 weeks
	Sham	-	4.8 ± 1.0	5.2 ± 1.3	5.2 ± 0.7	5.2 ± 0.9
	Model	-	4.8 ± 1.2 (0.9549)	7.0 ± 1.1^++ ^(0.0046)	7.5 ± 0.4^+++^	7.1 ± 0.7^+++^
LVIDd (mm)	ASI	0.1	5.0 ± 0.8 (0.6785)	6.9 ± 1.2 (0.9603)	6.9 ± 1.0 (0.1601)	6.5 ± 1.1 (0.1109)
	ASI	0.3	4.5 ± 1.2 (0.5850)	6.2 ± 1.9 (0.1946)	6.7 ± 1.7 (0.0547)	6.0 ± 0.9** (0.0057)
	ASI	1.0	4.7 ± 1.5 (0.7629)	6.4 ± 1.3 (0.3389)	6.5 ± 0.8* (0.0193)	6.0 ± 0.5**^$ ^(0.0046)
	Quinapril	1.0	5.1 ± 1.2 (0.6512)	6.6 ± 1.1 (0.5964)	6.7 ± 0.8 (0.0574)	6.7 ± 0.9 (0.3837)
	Sham	-	1.3 ± 0.4	1.6 ± 0.4	1.5 ± 0.3	1.8 ± 0.5
	Model	-	1.5 ± 0.5 (0.4268)	4.8 ± 1^+++^	5.6 ± 0.8^+++^	5.5 ± 0.8^+++^
LVIDs (mm)	ASI	0.1	1.4 ± 0.3 (0.8811)	4.9 ± 0.9 (0.8356)	4.5 ± 0.9* (0.0112)	4.7 ± 0.9* (0.0447)
	ASI	0.3	1.2 ± 0.4 (0.1827)	4.5 ± 1.6 (0.3955)	4.0 ± 1.6***	4.3 ± 1.0** (0.0027)
	ASI	1.0	1.4 ± 0.4 (0.6903)	4.4 ± 0.8 (0.3353)	3.7 ± 0.7***	3.8 ± 1.0***^$^
	Quinapril	1.0	1.6 ± 0.7 (0.5180)	4.6 ± 0.7 (0.6125)	3.9 ± 0.6***	4.8 ± 0.8 (0.0595)

Note: Values are mean ± SD in each group (*n *= 10). ^++^*P *< 0.01, ^+++^*P *< 0.001: statistical significance over the sham group (comparison of group means after ANOVA). *P < 0.05, **P < 0.01, ***P < 0.001: statistical significance over the model group (comparison of group means after ANOVA). ^$^*P *< 0.05: statistical significance between ASI (1.0 mg/kg/day) and Quinapril treatments (comparison of group means after ANOVA). Exact *P *values are available in brackets.

### Effects of ASI on haemodynamics

LVSP in the model group was 21% lower than that in the sham group, while LVEDP in the model group increased markedly, suggesting severe cardiac dysfunction in the CHF rats after coronary artery ligation (Table 4). ASI (at doses of 0.1 and 0.3 mg/kg/day) attenuated the decrease of LVSP, while LVEDP decreased 55.4% (*P *< 0.01) compared with the model group. The rats in the model group displayed higher wall stress of LV. ASI (1.0 mg/kg/day) significantly lowered the wall stress by 22% (*P *< 0.05) compared with the model group.

**Table 4:** Effects of ASI on LVSP, LVEDP and wall stress

Group	Dose (mg/kg/day)	LVSP (mmHg)	LVEDP (mmHg)	Wall stress (g/cm^2^)
Sham	-	111.8 ± 12.1	-0.7 ± 5.8	47.7 ± 17.6
Model	-	88.5 ± 14.0^+++^	13.9 ± 5.4^+++^	125.9 ± 28.0^+++^
ASI	0.1	101.4 ± 18.5* (0.0367)	10.6 ± 3.6 (0.1629)	120.4 ± 31.9 (0.6563)
ASI	0.3	101.6 ± 9.0* (0.0340)	9.8 ± 5.3 (0.0848)	108.3 ± 24.5 (0.1613)
ASI	1.0	100.2 ± 8.4^$$ ^(0.0571)	6.2 ± 4.2** (0.0018)	97.9 ± 29.8* (0.0283)
Quinapril	1.0	84.0 ± 15.5 (0.4566)	3.5 ± 6.3***	95.1 ± 31.7* (0.0167)

Note: Values are mean ± SD in each group (*n *= 10). ^+++^*P *< 0.001: statistical significance over the sham group (comparison of group means after ANOVA). *P < 0.05, **P < 0.01, ***P < 0.001: statistical significance over the model group (comparison of group means after ANOVA). ^$$^*P *< 0.01: statistical significance between ASI (1.0 mg/kg/day) and Quinapril treatments (comparison of group means after ANOVA). Exact *P *values are available in brackets.

### Effects of ASI on myocardial hypertrophy

LA, LV, RA and RV weight-to-tibial-length ratios decreased significantly after ASI (1.0 mg/kg/day) treatment compared with the model group (Table 5).

**Table 5:** Effects of ASI on myocardial hypertrophy

Group	Dose (mg/kg/day)	LA wt/TL (mg/mm)	LV wt/TL (mg/mm)	RA wt/TL (mg/mm)	RV wt/TL (mg/mm)	Tibial length (mm)	Body weight (g)
Sham	-	1.3 ± 0.6	20.3 ± 3.1	1.8 ± 0.6	5.5 ± 1.5	37.25 ± 1.23	290.2 ± 23.2
Model	-	2.8 ± 0.7^+++^	24.9 ± 2.1^+++^	2.6 ± 0.5^+++^	8.1 ± 1.5^+++^	38.20 ± 1.70	280.1 ± 24.3
		-		-		(0.1879)	(0.3439)
ASI	0.1	2.4 ± 0.4	22.7 ± 2.8	2.5 ± 0.4	7.1 ± 1.0	38.96 ± 1.28	271.2 ± 18.0
		(0.1855)	(0.0862)	(0.6751)	(0.1031)	(0.2723)	(0.4038)
ASI	0.3	2.5 ± 0.7	22.6 ± 4.0	2.5 ± 0.5	7.1 ± 1.2	37.23 ± 1.36	287.9 ± 31.6
		(0.2280)	(0.0796)	(0.7632)	(0.099)	(0.1811)	(0.4639)
ASI	1.0	2.1 ± 0.6*	21.0 ± 2.6**	2.0 ± 0.7*	6.1 ± 1.3**	37.89 ± 1.28	294.5 ± 15.7
		(0.015)	(0.0031)	(0.0186)	(0.0017)	(0.6827)	(0.1794)
Quinapril	1.0	2.0 ± 0.8**	22.0 ± 1.8*	2.0 ± 0.6*	7.2 ± 1.8	38.13 ± 2.27	280.1 ± 25.6
		(0.0076)	(0.0255)	(0.0223)	(0.1485)	(0.9434)	(1.0)

Note: Values are mean ± SD in each group (*n *= 10). ^+++^*P *< 0.001: statistical significance over the sham group (comparison of group means after ANOVA). *P < 0.05, **P < 0.01: statistical significance over the model group (comparison of group means after ANOVA). No statistical significance between ASI (1.0 mg/kg/day) and Quinapril treatments (comparison of group means after ANOVA). Exact *P *values are available in brackets.

### Effects of ASI on myocardial apoptosis and Bax

Assessment of apoptotic cell death was performed with *in situ *TUNEL staining of mycardial sections. Apoptosis was observed in the CHF rats with an increased level of Bax protein. Compared with the model group, both apoptotic cells and Bax proteins were reduced markedly after two weeks of ASI treatment (1.0 mg/kg/day) (Table 6).

**Table 6:** Effects of ASI on myocardial apoptosis and Bax

Group	Dose (mg/kg/day)	Apoptotic cell	Bax protein
Sham	-	3.0 ± 2.2	1.6 ± 1.3
Model	-	54.0 ± 10.2^+++^	30.2 ± 8.8^+++^
ASI	0.1	37.8 ± 8.6***	24.3 ± 7.1*
ASI	0.3	17.0 ± 8.4***	8.4 ± 5.3***
ASI	1.0	8.0 ± 5.6***	7.3 ± 3.4***
Quinapril	1.0	10.4 ± 6.4***	12.0 ± 5.5***

Note: Values are mean ± SD in each group (*n *= 10). ^+++^*P *< 0.001: statistical significance over the sham group (comparison of group means after ANOVA). *P < 0.05, ***P < 0.001: statistical significance over the model group (comparison of group means after ANOVA). No statistical significance between ASI (1.0 mg/kg/day) and Quinapril treatments (comparison of group means after ANOVA).

## Discussion

CHF is a serious outcome of MI. Many rats may die from serious ischemia after induction of acute MI by coronary artery ligation. However, rats suffering from slight ischemia may develop CHF. In the present study, surgery was performed on 141 rats, among which 48 rats died within 24 hours (34% mortality rate) and 61 rats developed CHF according to echocardiographic criteria for CHF [22]. The rats were dissected for the scar tissue at the end of experiments. Rats with coronary artery ligation had moderate infarcted area of 2.67 ± 0.39 (2.08–3.52) mg/mm. Rats in the sham group had no infarcted area.

Routinely used on humans, echocardiography is emerging as a noninvasive method for evaluating global systolic cardiac function and LV dimensions in rats [23-27]. In the present study, FS decreased 55.2% over baseline (*P *< 0.001); LVIDd and LVIDs increased markedly from 4.8 ± 1.2 mm and 1.5 ± 0.5 to 7.0 ± 1.1 mm and 4.8 ± 1.0 mm respectively (*P *< 0.001). These results are consistent with the findings by Oie *et al*. [22].

The mechanism of heart failure remains mostly unclear. The loss of cardiomyocytes may play an important role in causing heart failure [28]. Apoptosis is an important source for cardiomyocyte loss. There were 38.92 apoptotic cells per million myocardial cells in rats with heart failure, whereas the rate was only 2.21 cells per million myocardial cells in healthy rats [29].

A previous study showed that quinapril significantly suppressed the incidence of apoptotic myocytes around the necrotic region [30]. In the present study, quinapril significantly suppressed the incidence of apoptotic myocytes in the CHF rats. It was demonstrated that cardiomyocyte apoptosis and various genes associated with apoptosis were inhibited in mice with myocarditis after Astragaloside treatment [12]. In the present study, apoptotic myocytes significantly decreased in the CHF rats treated with ASI in a dose-dependent manner.

## Conclusion

The present study showed that ASI improved cardiac functions, inhibited compensatory hypertrophy of myocardial cells and lowered the number of apoptotic myocytes.

## Abbreviations

ASI: Astragaloside IV; Bax: Bcl-2 associated X protein; CHF: congestive heart failure; FS: fractional shortening; IVSs: interventricular septum thickness at end-systole; LA: left atrium; LV: left ventricle; LVIDd: left ventricle internal diameter in diastole; LVIDs: left ventricle internal diameter in systole; LVEDP: left ventricle end-diastolic pressure; -LVPWs: posterior wall thickness at end-systole; -LVSP: left ventricle systolic pressure; -MI: myocardial infarction; RA: right atrium; RV: right ventricle; SD: standard deviation; TL: tibial length; wt: weight.

## Competing interests

The authors declare that they have no competing interests.

## Authors' contributions

ZYZ, WTW, FW and KRZ drafted the manuscript and performed the experiments. WRX and YMH prepared reagents for the experiments. LDT supervised this work and revised the manuscript. All authors read and approved the final version of the manuscript.
